# The Tracy Technique: A Systems‐Based Paradigm Shift in Upper Face Neurotoxin Treatment

**DOI:** 10.1111/jocd.70867

**Published:** 2026-05-05

**Authors:** Benjamin Tracy, Kylie Moe

**Affiliations:** ^1^ The Injector Collective Lehi Utah USA

**Keywords:** aesthetic medicine, botulinum toxin, brow ptosis, frontalis muscle, injection technique, orbicularis oculi, upper face neurotoxin treatment

## Abstract

**Background:**

The Tracy Technique is a novel systems‐based method for upper face neurotoxin treatment. It challenges the traditional area‐based model by approaching the frontalis, glabella, and orbicularis oculi as a single dynamic complex.

**Objective:**

To describe the anatomical rationale and clinical application of the Tracy Technique and its ability to produce natural, balanced aesthetic outcomes.

**Methods:**

Patients were treated with onabotulinumtoxinA reconstituted with 2.5 mL bacteriostatic saline per 100 U vial (4 U per 0.1 mL). Injections were performed intramuscularly using a 32‐gauge, ½ inch needle at a 30‐degree angle, targeting the lower one‐third of the frontalis with lateral extension into the orbicularis oculi while sparing the middle third and minimizing treatment in the upper third. Clinical photographs were taken using the Alma IQ photo system under standardized conditions.

**Results:**

Three representative cases demonstrated effective rhytid reduction and maintenance of natural brow position without heaviness or ptosis. Both injectors and patients reported balanced, comfortable outcomes.

**Conclusion:**

The Tracy Technique reframes upper face neurotoxin treatment as a dynamic force balancing system. By treating both elevator and depressor muscles simultaneously, it optimizes aesthetic results and may establish a foundation for future evidence‐based refinement.

## 1 Introduction

Upper face expression arises from the interplay between one elevator muscle, the occipitofrontalis (frontalis), and several depressors, including the depressor supercilii, corrugator supercilii, procerus, and orbicularis oculi [1, 2]. Traditional injection paradigms acknowledge the relationships among these regions but continue to treat them as separate functional zones.

Anatomical and functional studies show that these muscles are interdigitated rather than independent. The lateral frontalis blends with the superior fibers of the orbicularis oculi, while its medial fibers overlap with the corrugator and procerus within a shared fascial plane [3, 4, 5]. Modulating one region, therefore, influences others, making isolated, zone‐based treatment an incomplete model for understanding upper face dynamics.

The lowest one‐third of the frontalis contributes the greatest lifting strength due to its dense vertical fiber orientation and direct dermal attachments [1, 6]. Despite this, it is often neglected in conventional practice because of the long‐standing caution against injecting within 1 cm of the brow or lateral to the mid‐pupillary line. Ironically, this caution stems from the very issue it seeks to prevent: brow descent caused by excessive weakening of the upper and middle frontalis before the lower fibers can compensate. This is due to the fact that when you inject into the upper and middle frontalis, you only inhibit the frontalis muscle, which can only have one outcome: the frontalis is weakened.

Preserving the middle third while precisely targeting the lower one‐third allows modulation of the most powerful frontalis fibers and direct influence on the depressor complex, effectively achieving a “two‐for‐one” result from a single injection point. This balance of elevation and relaxation forms the anatomical foundation of the Tracy Technique.

An additional and often overlooked anatomical consideration is the integration of the orbicularis oculi into the glabellar complex. The orbicularis oculi, particularly its orbital fibers, originates from the nasal part of the frontal bone, the frontal process of the maxilla, and the medial palpebral ligament and interdigitates with adjacent glabellar musculature [4, 7, 8]. When untreated, its lateral fibers tend to pull medially to compensate for weakened corrugator and procerus muscles, producing incomplete or suboptimal aesthetic results. The Tracy Technique accounts for this by incorporating targeted treatment of the lateral orbicularis oculi, which reduces glabellar “11” lines and improves overall patient satisfaction.

## 2 Methods

All treatments were performed using onabotulinumtoxinA (Botox Cosmetic; Allergan Aesthetics, Irvine, CA) reconstituted with 2.5 mL bacteriostatic saline per 100 U vial (4 U per 0.1 mL). All doses are reported as onabotulinumtoxinA equivalents.

### 2.1 Photography and Image Analysis

All pre‐treatment and post‐treatment clinical photographs were obtained using the Alma IQ photo system (Alma Lasers, Israel) under standardized lighting, camera settings, and patient positioning. Each subject was photographed in neutral, raised brow, and furrowed expressions. Patients maintained a neutral head position and identical expressions for each capture. Minor variations in gaze or facial tension may account for subtle differences in eyelid or brow position. These represent normal variability and not true ptosis or asymmetry.

### 2.2 Informed Consent

All patients provided written informed consent prior to treatment and photography. Each participant was informed of the purpose of the technique demonstration, the nature of the injections, potential benefits and risks, and the intended use of their de‐identified photographs for educational and publication purposes. Participation was voluntary, and patients were assured that refusal or withdrawal would not affect their clinical care.

## 3 Technique Description

The Tracy Technique reframes the upper face neurotoxin strategy by viewing the region as a continuous functional system rather than a collection of isolated compartments. Treatment begins with an assessment of baseline expression, muscle tone, and any asymmetries in brow position or frontalis activity.

Injections are concentrated in the lower one‐third of the frontalis, aligned with the strongest elevating fibers. This region provides the primary lifting force and counterbalances the glabellar and orbital depressors. By focusing treatment here while leaving the mid‐frontal area untreated, injectors achieve a “two‐for‐one” relaxation effect that influences the glabellar and orbital complexes through functional interplay. The result is a net lifting potential on brow position, as preserved mid‐frontalis activity creates an elevator dominance relative to the brow depressors.

Traditional “no‐go” zones, particularly those near or lateral to the mid‐pupillary line and within 1 cm of the brows, are reconsidered within this systems‐based framework. Carefully placed injections lateral to the mid‐pupillary line, extending toward the arch and above the lateral canthus, can reduce overactivity while maintaining lateral brow lift. The upper one‐third of the frontalis may receive limited microinjections to ensure uniformity and prevent visible demarcations, maintaining dynamic balance rather than creating global weakness.

**FIGURE 1:**
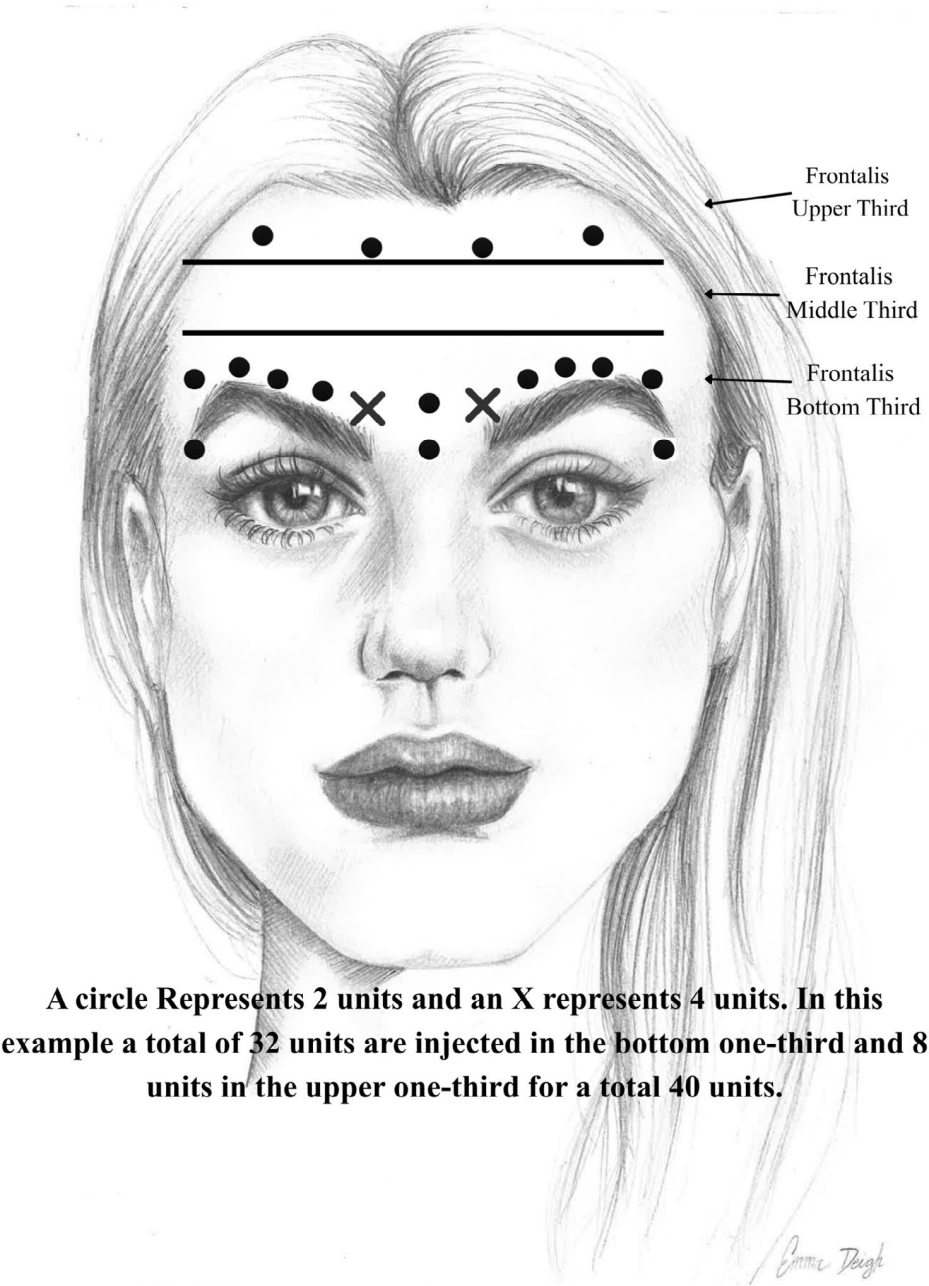
Injection mapping demonstrating lower‐third focus and lateral extension pattern.

All injections were intramuscular using a 32‐gauge, ½ inch needle on a 1‐mL zero loss slip tip syringe (BD Ultra Fine). The needle was introduced at a 30‐degree angle and directed medially or laterally away from the mid‐pupillary line (Figure 1).

## 4 Illustrative Cases

### 4.1 Case 1

**FIGURE 2:**
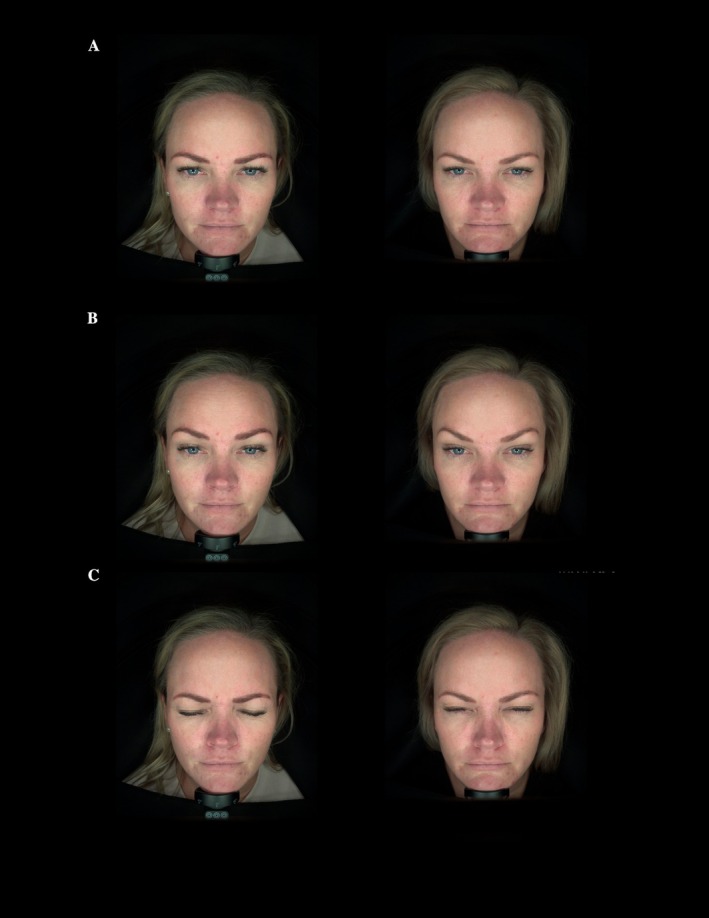
Standardized before‐and‐after images at (A) rest, (B) elevation, (C) frown.

A 37‐year‐old woman received 40 units across the lower one‐third of the forehead and glabella complex, extending laterally below the arch but above the lateral canthus, and 10 units in the upper one‐third of the frontalis. At 2 weeks post‐treatment, she demonstrated improved brow symmetry, reduced glabellar hyperactivity, and maintained natural frontalis elevation (Figure 2).

### 4.2 Case 2

**FIGURE 3:**
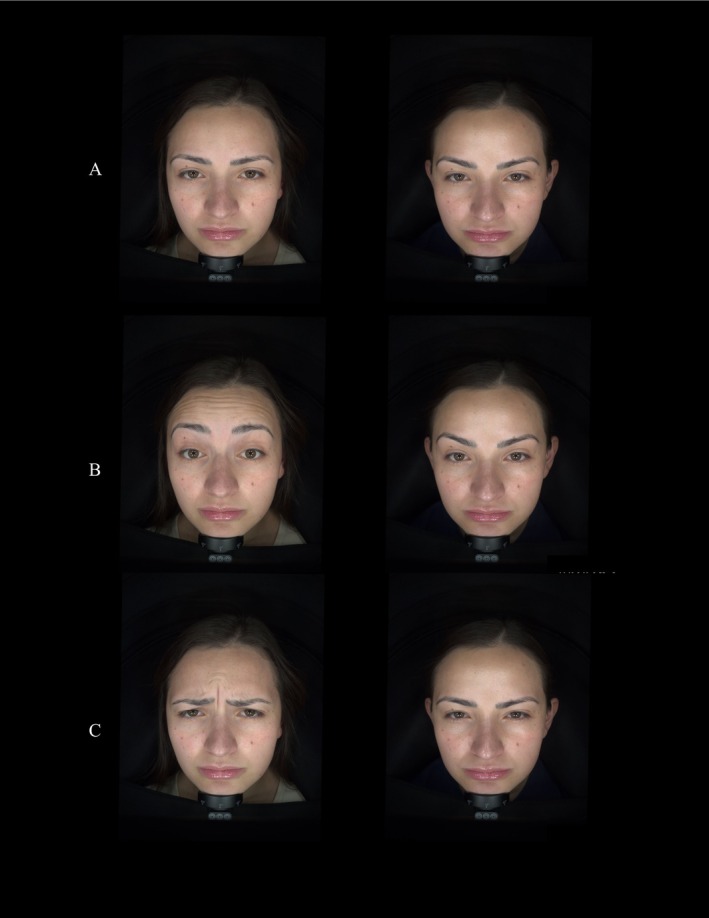
Standardized before‐and‐after images at (A) rest, (B) elevation, (C) frown.

A 32‐year‐old woman received 32 units in the lower one‐third of the frontalis and glabella and 10 units in the upper one‐third. Follow‐up showed sustained relaxation of the central brow with preserved expressive movement. Both injector and patient assessments confirmed natural expression, aesthetic satisfaction, and no adverse events (Figure 3).

### 4.3 Case 3

A 40‐year‐old woman received 36 units in the lower one‐third of the frontalis and glabella and 10 units in the upper one‐third. At 2‐week follow‐up, the patient demonstrated smoothing of dynamic lines, preservation of lift, and an overall balanced, rested appearance without heaviness. She noted that her treatment seemed to start taking effect quicker than she normally experiences and that she did not experience brow drop or heaviness. No adverse events were reported.

**FIGURE 4:**
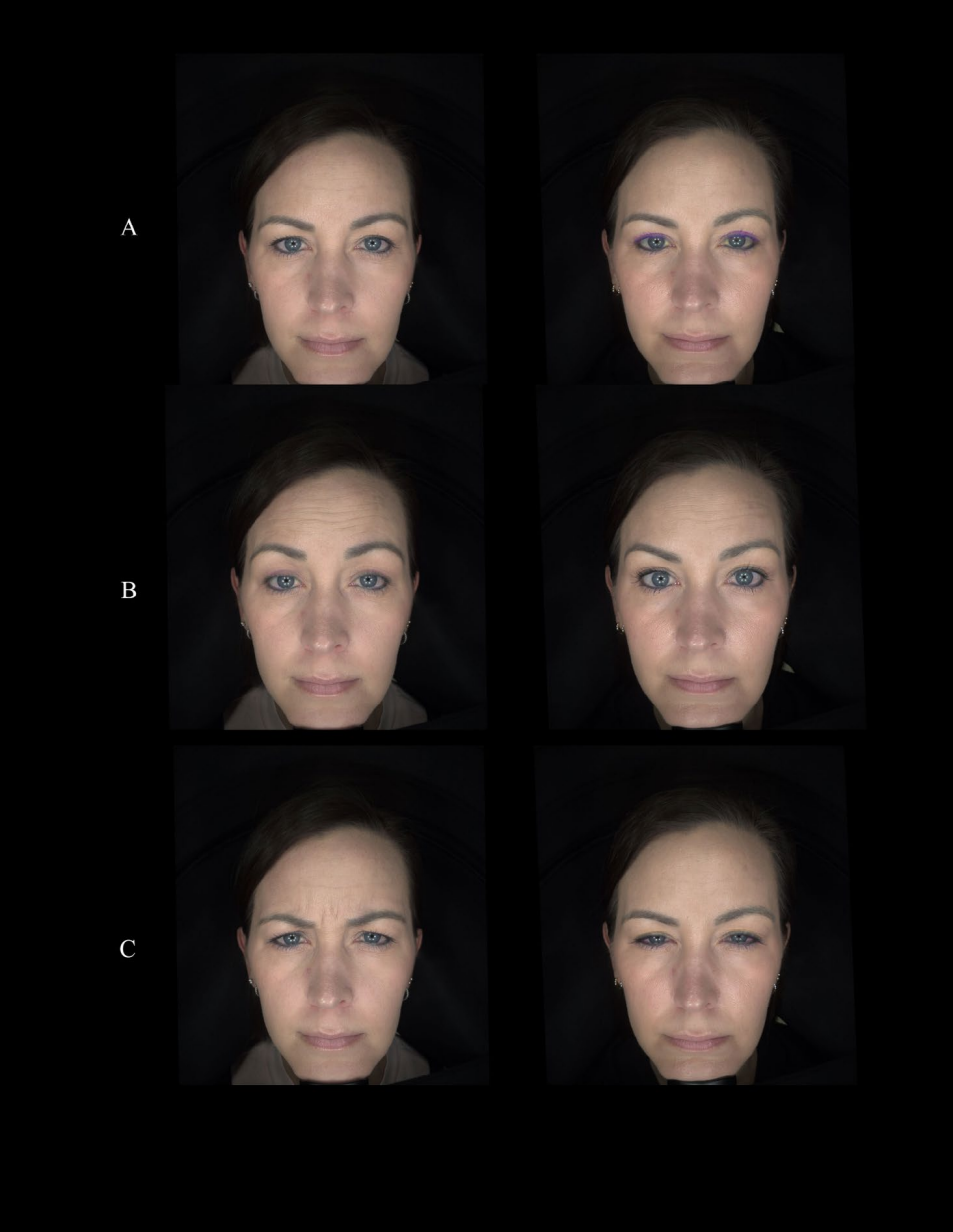
Standardized before‐and‐after images at (A) rest, (B) elevation, (C) frown.

All patients tolerated treatment well, and no adverse events were reported (Figure 4).

## 5 Discussion

The Tracy Technique challenges the long‐standing approach of treating the upper face in isolated regions. Conventional forehead‐only protocols weaken the frontalis as a whole, removing the ability to address the lower portion of the muscle that is essential for balancing the downward pull of the depressors. As a result, injectors often avoid the lateral lower third entirely, fearing brow descent.

By reversing this paradigm and focusing on the lower one‐third and glabellar complex while preserving mid‐frontal strength, this method restores balance between opposing muscle groups. It recognizes that the glabella and frontalis are functionally linked rather than separate units.

A further advantage of this approach is that it accounts for the orbicularis oculi as an integral part of the glabellar complex. Traditional techniques often overlook this relationship. The orbicularis oculi originates from the nasal part of the frontal bone and inserts into the surrounding soft tissues of the medial brow and glabella. When left untreated, its lateral fibers tend to pull medially to compensate for weakened corrugator and procerus muscles, leading to incomplete relaxation and less satisfying outcomes. By incorporating targeted treatment of the lateral orbicularis oculi, the Tracy Technique minimizes this compensatory pull, reduces glabellar “11” lines, and improves overall patient satisfaction.

This systems‐based approach aligns with current literature emphasizing that natural, balanced results rely on coordinated modulation of both elevators and depressors [1, 9, 10]. It replaces excessive caution with anatomical precision and logic, expanding the safe and effective boundaries of neurotoxin treatment.

## 6 Future Directions

Future studies should include randomized, double‐blind controlled trials comparing the Tracy Technique with conventional forehead–glabellar protocols. Key outcome measures should include wrinkle severity scales, brow height symmetry, patient satisfaction indices, and electromyographic mapping to quantify muscular balance. These studies will further validate the systems‐based model and refine dosing parameters and injection depth for individualized treatment.

## 7 Conclusion

The Tracy Technique represents a shift from zone‐based paralysis to functional modulation, treating the upper face as an interconnected system. By prioritizing the lower frontalis and preserving mid‐frontal strength, injectors can achieve smoother, natural results while reducing the risk of brow descent. This technique emphasizes that effective neurotoxin artistry arises from viewing facial anatomy not as fixed compartments but as a coordinated, dynamic network.

## Author Contributions

Conceptualization and methodology: **Benjamin Tracy**. Clinical execution and injector contribution: **Kylie Moe**. All authors reviewed and approved the final manuscript and agree to be accountable for all aspects of the work.

## Funding

The authors have nothing to report.

## Ethics Statement

The authors confirm adherence to the ethical policies of the *Journal of Cosmetic Dermatology*. No IRB approval was required as this work presents a retrospective description of standard clinical technique.

## Consent

All patients provided written informed consent for treatment and for publication of anonymized images.

## Conflicts of Interest

The authors declare no conflicts of interest.

## Data Availability

Data supporting the findings of this article are available from the corresponding author upon reasonable request.
